# Development and optimization of curcumin analog nano-bilosomes using 2^1^.3^1^ full factorial design for anti-tumor profiles improvement in human hepatocellular carcinoma: *in-vitro* evaluation, *in-vivo* safety assay

**DOI:** 10.1080/10717544.2022.2044938

**Published:** 2022-03-04

**Authors:** Haidy Abbas, Yasmin A. El-Feky, Majid Mohammad Al-Sawahli, Nehal M. EL-Deeb, Hala Bakr El-Nassan, Mariam Zewail

**Affiliations:** a Pharmaceutics Department, Faculty of Pharmacy, Damanhour University, Damanhur, Egypt; b Department of Pharmaceutics, Faculty of Pharmacy, Modern University for Technology and Information (MTI), Cairo, Egypt; c Department of Pharmaceutical Technology, Faculty of Pharmacy, Kafr Elsheikh University, Kafr Elsheikh, Egypt; d Department of Pharmaceutics, College of Pharmacy, The Islamic University, Najaf, Iraq; e Biopharmaceutical Products Research Department, Genetic Engineering and Biotechnology Research Institute, City of Scientific Research and Technological Applications, Alexandria, Egypt; f Pharmaceutical Organic Chemistry Department, Faculty of Pharmacy, Cairo University, Cairo, Egypt

**Keywords:** Curcumin, 3,5-bis(4-bromobenzylidene)-1-propanoylpiperidin-4-one, hepatocellular carcinoma, nano-bilosomes, bile salts

## Abstract

Curcumin (CU) is a natural polyphenolic phytoingredient. CU has anti-inflammatory, anti-oxidant, and anticancer activities. The poor solubility, bioavailability, and stability of CU diminish its clinical application. Hence, structural modification of CU is highly recommended. The CU analog; 3,5-bis(4-bromobenzylidene)-1-propanoylpiperidin-4-one (PIP) exhibited high stability, safety, and more potent antiproliferative activity against hepatocellular carcinoma. In the present study, nano-bilosomes (BLs) were formulated to augment PIP delivery and enhance its solubility. A 2^1^.3^1^ full factorial design was adopted to prepare the synthesized PIP-loaded BLs. Optimized F4 showed a biphasic release pattern extended over 24 h, with EE%, ZP, and PS of 90.21 ± 1.0%, −27.05 ± 1.08 mV, and 111.68 ± 1.4 nm. PIP-loaded BLs were tested for safety against a non-cancerous cell line (Wi-38) and for anticancer activity against the Huh-7 human hepatocellular carcinoma cells and compared to the standard anticancer drug doxorubicin (Dox). The anticancer selectivity index of PIP-loaded BLs recorded 420.55 against Huh-7 liver cancer cells, markedly higher than a CU suspension (18.959) or the Dox (20.82). The antiproliferative activity of nano-encapsulated PIP was roughly equivalent to Dox. PIP-loaded BLs, showed enhanced drug solubility, and enhanced anticancer effect, with lower toxicity and higher selectivity against Huh-7 liver cancer cells, compared to the parent CU.

## 1. Introduction

Hepatocellular carcinoma is the most common primary liver malignancy, which is responsible for about 75–85% of primary liver cancers. It can be ranked as the sixth most widespread cancer and the second major cause of cancer-related deaths around the world (Rashed et al., 2020). Although numerous trials were adopted for preventing, assaying techniques, and new technologies in both diagnosis and treatment, the mortality rates continue to rise. The highest incidence rates may be attributed to the greater prevalence of hepatitis C virus infection, which greatly increases hepatocellular carcinoma risk (Abudeif, 2019; Rashed et al., 2020). Liver cancer is often poorly responsive to chemotherapeutic treatment and prone to the development of drug resistance (Abudeif, 2019) necessitating a continued search for safer and more effective alternatives.

**Figure 1.**
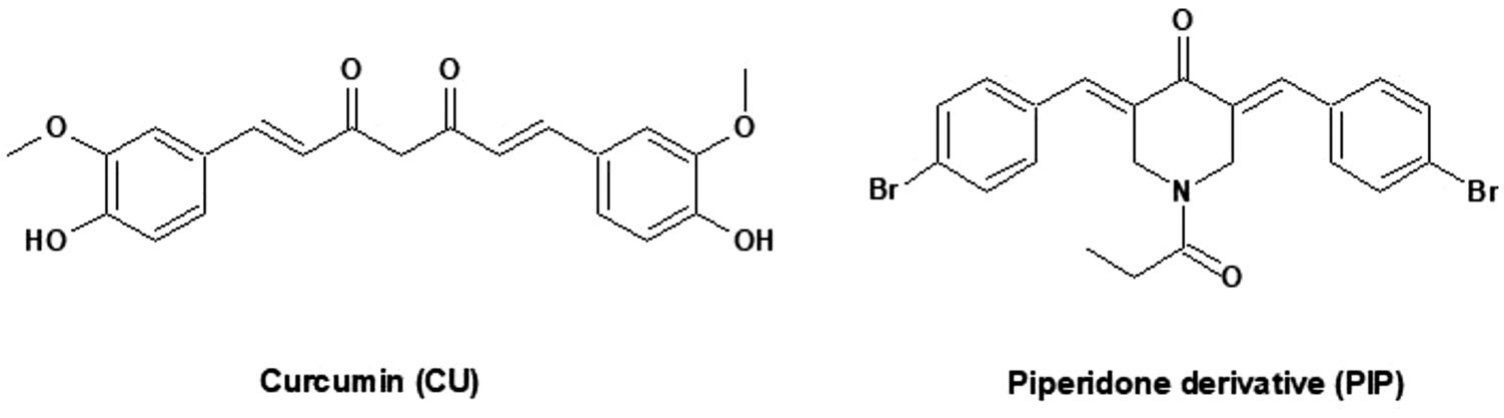
Chemical structure of curcumin (CU) and piperidone (PIP).

Curcumin (CU) (diferuloylmethane, 1,7-bis(4-hydroxy-3-methoxyphenyl)-1,6-heptadien-3,5-dione, Figure 1) which is shown in Figure 1, is one of the most abundant polyphenols found in turmeric and a major bioactive component of turmeric extracts (Chen et al., 2018). CU has a very low water solubility which might be associated with poor bioavailability. Documented pharmacological effects of CU include anti-inflammatory, antiviral, antioxidant, and antibacterial activities. Further, various CU extracts have shown therapeutic efficacy in animal models of diabetes and Alzheimer’s disease (El-Nassan, 2014; Kim & Clifton, 2018). In addition, CU has strong cytotoxic potential against certain cells (Teiten et al., 2010), as well as broad-spectrum anticancer activities, including against liver cancer cells (Mehta et al., 1997; Walters et al., 2008; Bimonte et al., 2016; Shi et al., 2017; Liu & Ho, 2018). While the underlying molecular mechanisms are uncertain in many cases, CU is known to modulate multiple signaling pathways and to protect cells against chemotoxicity through antioxidant and anti-inflammatory activities (López‐Lázaro, 2008; Darvesh et al., 2012; Ren et al., 2018).

Despite numerous biological activities, direct therapeutic applications of CU are limited by several innate physicochemical properties, including low solubility (CU is practically insoluble in water), low bioavailability, and various interactions with other compounds. In addition, CU is unstable as it is reactive in the presence of metallic ions, oxygen, light, and heat (Priyadarsini, 2014; Chen et al., 2018). This instability may be related to the ease of tautomeric conversion of the central conjugated β-diketone moiety, especially in alkaline medium. In addition, the β-diketone moiety of CU is the specific substrate for a series of aldo-keto reductases and consequently CU is rapidly metabolized (Teiten et al., 2010).

Previous studies reported that CU exerts anticancer effects at concentrations ranging from 5 to 50 μM upon exposure for several hours (Syng-Ai et al., 2004; Cao et al., 2012). As a result of reduced solubility and low bioavailability, it was difficult to obtain a satisfying CU concentration range in target tissues after CU absorption from the gastrointestinal tract (Teiten et al., 2010). Several approaches have been adopted to overcome these limitations and promote more effective oral CU administration, including various structural modifications (Youssef et al., 2004; Modzelewska et al., 2006; Liang et al., 2009; Shaikh et al., 2009; Kim et al., 2012) to yield more potent CU analogs with better pharmacokinetic profiles. Replacing the β-diketone moiety with a monocarbonylenone moiety is a suggested structural modification to inhibit tautomerism.

Recently, a new series of 3,5-bisbenzylidenepiperidone derivatives as potent anti-tumor agents were developed. Among them, 3,5-bis(4-bromobenzylidene)-1-propanoylpiperidin-4-one(PIP) (Figure 1) showed highly potent anticancer activity (Teiten et al., 2010). Thus, PIP was tested in the NCI Developmental Therapeutic Program on nine tumor panels (CNS, leukemia, colon, renal, breast, melanoma, prostate, lung, and ovarian cancer) and displayed an MG-MID GI_50_of 0.35 µM, a TGI of 1.62 µM, and an LC_50_ of 9.12 µM. Furthermore, PIP exhibited broad-spectrum antiproliferative potential with GI_50_ values below 1 µM in 81% of the tested cell lines and was 21 times more potent than CU (Teiten et al., 2010). However, the effect of PIP on liver cancer cell lines has not been tested. Despite potent antiproliferative efficacy against several cancer cells lines, the pharmacokinetic parameters of PIP are still not optimal for oral drug administration, including high lipophilicity and poor expected water solubility (log *P* = 5.77). Nano-encapsulation is another reported approach for improving the solubility, stability, and bioavailability of CU (Suwannateep et al., 2011). Several CU-loaded nano-carriers have been described, such as nanocapsules (Suwantong et al., 2007), nanofibers (Spicer et al., 2001; Spicer, 2005; Wang et al., 2017), cubosomes (Spicer et al., 2001; Spicer, 2005; Tiyaboonchai et al., 2007) solid lipid nanoparticles (Rahman et al., 2014; Tavano et al., 2014; Ramalingam et al., 2016), niosomes (Song et al., 2011), polymeric micelles (Sawant & Bamane, 2018), inorganic nanoparticles (Samrot et al., 2018), polymeric nanoparticles (Saengkrit et al., 2014), and liposomes (Conacher et al., 2001).

Bilosomes (BLs) are unconventional colloidal distribution for bile salts (BS) to be engineered and inserted within liposomes. BLs are nano-sized structures that enclose surfactants and bile salts which suggest functional efficiency in oral drug delivery. BL represents new platforms of nano-vesicular carriers for drug delivery, that were first developed in the innovative work by Conacher et al. (2001). The involvement of BS increases liposomal resilience. They are more flexible, elastic, and extremely deformable than conventional liposomes. BLs efficiency for drug delivery has been confirmed in previous literature, they can improve antigen effectiveness, which is poor when injected, hence, they are healthy and efficient relative to conventional vaccines, bile salts present in the lipid bilayers of BL enhance their resistance against gastrointestinal (GI) bile salts and enzymes, therefore, support protection of the entrapped vaccine against the hostile environment of the GI tract, also, are noninvasive and deliver excellent patient support and compliance. Additionally, Bile salts are absorbed through apical sodium-dependent bile acid transporter (ASBT) in the GIT, which can act as an enhancer in oral delivery (Al-Mahallawi et al., 2015; Aburahma, 2016; Waglewska et al., 2020).

The aim of the current study was to formulate and evaluate the synthesized PIP having an improved anticancer potential compared to CU against hepatocellular carcinoma cells, in BLs adopting 2^1^.3^1^ full factorial design. The obtained BLs are expected to have assimilation-enhancing ability that can increase the drug water solubility and hence, can increase the drug absorption and protect it from the expected enzymatic degradation into GIT. In addition, bile salts can act as solubilizing and permeation enhancing agents which can also improve the drug oral absorption. The prepared formulations were evaluated and optimized to obtain the formula giving the highest desirability according to different chosen dependent variables. This work can be considered the first report of encapsulating curcumin analog (PIP) with enhanced anticancer activity in BLs to combine the advantages of the superior pharmacological activity of CU analog along with the numerous advantages of BLs in terms of BLs' ability to increase the encapsulated drug solubility, stability, permeation across biological barriers and consequently drug bioavailability and pharmacological effects are further improved.

## 2. Materials and methods

Phosphatidylcholine (PDC), phosphatidyl serine (PDS), pluronic (P123), curcumin (CU), and sodium cholate hydrate (SC) were purchased from Sigma-Aldrich (Germany). Cholesterol (CH) was provided by CRODA Inc. (East Yorkshire, England). All other chemicals and reagents used in this study were of analytical grade.

### 2.1. Synthesis of 3,5-bis(4-bromobenzylidene)-1-propanoylpiperidin-4-one (PIP)

A mixture of 3,5-bis(4-bromobenzylidene)-piperidin-4-one(5 mmol) and propionic anhydride (1.95 g, 5 mmol) in toluene (50 mL) was heated under reflux for 3 h. The solvent was evaporated under reduced pressure and the solid formed was filtered, dried, and crystallized from ethanol (El-Nassan, 2014). Physical and chemical properties of the crystal were as follows: yield57%; mp 185–186 °C (El-Nassan, 2014); IR (cm^−1^) 2935, 2846 (CH aliphatic), 1670, 1654 (C = O); ^1^H NMR (DMSO-d_6_) δ ppm 0.80 (t, 3H, *J* = 7.4 Hz, CH_2_CH_3_), 2.14 (q, 2H, *J* = 7.4 Hz, CH_2_CH_3_), 4.78 (br s, 4H, two CH_2_), 7.50 (d, 4H, *J* = 8.1 Hz, Ar–H), 7.64 (d, 4H, *J* = 8.1 Hz, Ar–H), 7.72 (s, 2H, C = CH); ^13 ^C NMR (75 MHz, DMSO-d_6_) δ ppm 8.8, 25.0 (CH_3_CH_2_), 42.3, 45.8 (C-2, C-6), 123.0, 130.7, 131.7, 132.5 (aromatic carbons), 133.2 (C-3, C-5), 134.8 (CH=), 171.7, 185.9 (C = O).

### 2.2. Experimental design

Synthesized PIP-loaded BLs were prepared to adopt a full factorial design (2^1^.3^1^), to estimate and optimize the effect of the variant selected independent variables according to preliminary studies. At this design, two factors upon different levels were assessed. As presented in Table 1, the phospholipid type (X_1_) was upon two levels phosphatidyl choline (PDC) or phosphatidyl serine (PDS), while, (X_2_) the sodium cholate concentration (SC) was upon three levels 0.1, 0.3, and 0.6%. The experimental trials were performed with all possible combinations for the formulation of loaded BLs (Table 2). All the data were calculated three times and expressed as mean ± standard deviation (SD). The entrapment efficiency (EE%, R_1_), zeta potential, ***ζ*** (ZP, R_2_), and particle size (PS, R_3_), were selected as the measured dependent variables (responses). To determine the relationship between independent variables and dependent variables, three-dimensional (3D) surface plots and one-factor effect were plotted. The desirability function of PS was at the minimum level, while the EE% and ZP were at the highest level. Design-Expert^®^ software version 8 (Stat-Ease, Inc., Minneapolis, MN, USA) was utilized to analyze experimental data to investigate independently the effects of these variables. Analysis of variance (ANOVA) was proceeded to determine the significance of variant variables.

**Table 1.** 2^1^.3^1^ full factorial design variables and constraints.

Independent variables	Levels			
	−1	0	+1	Constrains
X_1_: phospholipid type	PDC	–	PDS	In range
X_2_: SC concentration	0.1%	0.3%	0.6%	In range
Dependent variables				
R_1_: EE (%)				Maximize
R_2_: ZP (mV)				Maximize
R_3_: PS (nm)				Minimize

PDC: phosphatidyl choline; PDS: phosphatidyl serine; SC: sodium cholate; EE%: entrapment efficiency; ZP: zeta potential; PS: particle size.

**Table 2.** Independent variables and measured responses for the 2^1^.3^1^ full factorial experimental design of PIP loaded BLs.

Formulation code	X_1_ phospholipid type	X_2_ SC conc. (%)	R_1_ EE (%)	R_2_ ZP (mV)	R_3_ PS (nm)	PDI
F1	PDC	0.1	96.98 ± 1.4	−15.13 ± 2.03	168.17 ± 0.08	0.008 ± 0.023
F2	PDC	0.3	91.04 ± 2.6	−21.20 ± 1.56	200.23 ± 1.05	0.005 ± 0.072
F3	PDC	0.6	87.33 ± 2.45	−28.22 ± 1.14	265.2 ± 0.32	0.002 ± 0.112
F4	PDS	0.1	90.21 ± 1	−27.05 ± 1.08	111.68 ± 1.4	0.006 ± 0.023
F5	PDS	0.3	86.11 ± 0.94	−30.96 ± 1.12	141.99 ± 0.98	0.004 ± 0.091
F6	PDS	0.6	81.08 ± 1.7	−35.11 ± 1.56	206.78 ± 1.2	0.003 ± 0.012

Cholesterol (CH) and pluronic 123 (P 123) were kept constant in all formulae at 0.3 and 0.6%, respectively.

### 2.3. Preparation of PIP loaded BLs

Synthesized PIP-loaded BLs were formulated using the thin film hydration technique described by Waglewska et al. (2020). Briefly, 0.05 mg/ml PIP, pluronic 123 (P123, 0.6%), cholesterol (CH, 0.3%), and PDC or PDS (1%) were solubilized by the use of an ultrasonic bath sonicator (Ultrasonic bath sonicator, Model SH 150-41; USA) for 10 min in 10 mL chloroform in a round bottom flask. The obtained organic solution was subjected to evaporation at 40 °C under reduced pressure using a rotary evaporator (Rotavapor, Heidolph VV 2000; Heidolph Instruments, Kehlheim, Germany) for 30 min till obtaining a dry and thin film. The film formed by evaporation was left overnight to ensure complete evaporation of residual organic and then rehydrated in 10 ml distilled water containing SC. The resultant dispersion was magnetically stirred for 2 h to generate a dispersion of PIP-loaded BLs. To reduce particle size (PS) of the obtained dispersion of BLs, the dispersion was ultrasonicated for 5 min (Bandelin, Berlin, Germany). The prepared synthesized PIP loaded BLs dispersion was kept at 4 °C until use.

### 2.4. Characterization of the prepared PIP loaded BLs

#### 2.4.1. Particle size (PS), zeta potential (ZP, ζ), and polydispersity index (PDI), measurement

PS, PDI, and ZP (ζ) of prepared BLs were measured using a Malvern zeta sizer Nano ZS (Malvern Instruments, Malvern, UK). Samples (*n* = 3) of each formulation were enough diluted in deionized water to be measured.

#### 2.4.2. Entrapment efficiency percentage (EE%)

Entrapment efficiency was estimated by the direct method. BLs were sonicated in ethanol for 10 min and filtered through a syringe filter (pore size: 0.4 μm) (Millex-LG, Millipore Co., USA). The amount of PIP entrapped within BLs was estimated using a Shimadzu UV spectrophotometer (2401/PC Japan) at 425 nm (El-Nassan, 2014; Van Nong et al., 2016) The EE% was calculated using the following equation (Equation 1):

(1) $$\text{EE \%}=\frac{\mathrm{Amount of encapsulted drug}}{\mathrm{Total amount of drug}}\times 100.$$

#### 2.4.3. *In-vitro* drug release and release kinetics

*In-vitro* drug release was measured using the dialysis bag diffusion technique. Briefly, a sample of BLs dispersion containing 10 mg PIP was placed in the cellulose dialysis bag and submerged in up to 15 ml of 0.1 M PBS (pH 7.4), 0.1% tween 20 was added to maintain sink condition. At predetermined time intervals, 2-ml samples of the receiver medium were withdrawn and replaced by an equivalent fresh medium volume to maintain both constant volume and sink conditions. The amount of PIP in receiver medium samples was determined by UV spectrophotometry at 425 nm (Abbas et al., 2018; El-Telbany et al., 2021). Release data from CU, PIP suspensions, and different BLs formulations were fitted to zero, first-order, and Higuchi equations using DD solver software.

#### 2.4.4. Morphological examination

The surface morphology of synthesized PIP-loaded BLs, including shape and surface was evaluated by transmission electron microscopy, TEM (JEM-2100; JEOL, Japan). The sample was diluted with double distilled water and then placed on a copper grid that was coated with carbon (El-Telbany et al., 2021).

#### 2.4.5. Safety assay

The maximum nontoxic dose to non-cancerous Wi-38 cells was determined by MTS cell viability assays. A cell suspension (6 × 104 cell/ml) was seeded at 100 µl per well in 96-well plates and incubated at 37 °C in humidified 5% CO_2_ for 24 h. The culture medium was discarded and replaced by either 100 µl of fresh medium (negative control) or medium containing different concentrations of encapsulated PIP. Cells were incubated under the same growth conditions for 3 days, and then viable cell numbers were estimated using a colorimetric MTS assay Kit according to the manufacturer’s instructions.

#### 2.4.6. *In-vitro* anticancer activity of CU and PIP loaded BLs

The anticancer effects of CU suspension and PIP-loaded BLs were tested against the Huh-7 liver cancer cell line and compared to the standard anticancer drug (doxorubicin). Anticancer effects and IC_50_ values were quantified using MTS assay as described.

#### 2.4.7. Selectivity index (SI)

The selectivity index was calculated by the method of Koch et al. (2005). The SI values were calculated according to the following equation (Equation 2):

(2) $$SI=\frac{{IC}_{50nc\;}}{{IC}_{50cc\;}}$$

Where, IC_50nc_ refers to the IC_50_ value of the tested compound in normal cells while IC_50cc_ refers to the IC_50_ of the tested compound on a cancer cell line.

#### 2.4.8. Cu and PIP cellular uptake by Huh-7 cells

After determination of the 24-h IC_50_ values for CU and PIP, the treated Huh-7 liver cancer cells were harvested using trypsin, and uptake was quantified by flow cytometry (FACSVerse, BD Biosciences). The live single-cell population was gated in a plot of FSC *vs.* SSC, and a histogram from the FITC channel for the single-cell population was gained and analyzed using FlowJo (version X, FlowJo LLC).

#### 2.4.9. Quantification of the induced ROS using oxidized DCFDA and flow-cytometry

Reactive oxygen species (ROS) and oxidative damage are thought to play an important role in many human diseases. Using cell-permeable fluorescent and chemiluminescent probes, 2′-7′-Dichlorodihydrofluorescein diacetate (DCFH-DA), the induced ROS in CU and PIP-treated cells were quantified by flow cytometry (Al-Madboly et al., 2020) and compared with and lipopolysaccharides (LPS)-induced PBMCs cells (positive control). Briefly, peripheral blood mononuclear cells were isolated from whole human blood using gradient separation by Ficoll-PagueTM Plus (MP Biomedicals, France) as reported (Lohr et al., 1995) and used as a model to quantify the induced ROS. CU and PIP-treated cells, LPS -induced and the control cells were incubated with DCFH-DA at a final working concentration of 10 μM dyes for 30 min at 37 °C and 5% CO_2_. At the end of incubation, the collected cells were washed with prewarmed PBS and suspended in FAC buffer solution. The intensity of fluorescence was examined by flow cytometry (parts flowcytometry), The redox state of the sample could be monitored by detecting the increase in fluorescence that could be measured at 530 nm when the sample is excited at 485 nm.

#### 2.4.10. Statistical analysis

Data are presented as mean ± *SD*. Two means were compared by Student’s *t*-test and three or more groups is meant by one-way analysis of variance (ANOVA) with X tests for pair-wise comparisons. A *p* < .05 (two-tailed) was considered significant. All statistical calculations were conducted using Graph Pad Prism 7.

## 3. Results and discussion

### 3.1. Preparation of PIP loaded BLs

Miere et al. (2021) stated the advantages of preparing liposomes using a mixture of phospholipids and cholesterol, as this combination proved to increase liposomal membrane permeability, and hence, increase the binding of liposomes with cells *in vivo*. Addition of bile salts to the aforementioned components to prepare BLs has proven to enhance drug solubility, stability, and permeation through gastrointestinal barriers. Bile salts are biocompatible with no toxicity profile. They can act as solubilizing and permeation-enhancing agent. Sodium cholate (SC) is one of the most commonly used bile salts due to its nontoxic nature and high permeation enhancing capacity and hence it was utilized to prepare PIP BLs (Zafar et al., 2021).

The choice of P123 as a surfactant in the present study was based on several known advantages, including low immunogenicity, lack of irritation upon topical or subcutaneous application, and ready elimination by the kidneys. Moreover, the presence of polyethylene oxide groups in its structure can reduce BLs recognition by the mononuclear phagocytic system, further enhancing stability (Waglewska et al., 2020). Furthermore, it was reported that P123 is capable of restoring the sensitivity of multidrug-resistant tumor cells to anticancer agents (Zhao & Zhang, 2017; Waglewska et al., 2020).

### 3.2. Characterization of the prepared PIP loaded BLs

The colloidal characteristics of PIP-loaded BLs are summarized in Table 2. The PS of the prepared BLs ranged from 111.68 ± 1.4 to 265.2 ± 0.32 nm. The low PDI value 0.112 ± 0.03 to 0.136 ± 0.08 (too small values), confirmed that the size of the prepared BL was uniform. The optimum value for PDI is <0.3, which will be accepted to reflect the homogeneous distribution of the nanoparticles. The PDI should be as low as possible (ideally, nearly zero), hence, the size distribution is unimodal (El-Telbany et al., 2021). Nanocarriers PS is a crucial determinant of the drug fate *in-vivo*, as smaller nanocarriers in size permit greater trans-cellular uptake than larger ones. In other words, the small unilamellar vesicles appear to increase partitioning to the bone marrow. Also, they increase the longevity of vesicles in circulation (Borborema et al., 2016). It was reported that nanocarriers smaller than 500 nm can penetrate the bloodstream efficiently (Priya & Iyer, 2020). ZP is an important indicator of formulation stability as a higher surface charge produces repulsive forces that prevent particle coalescence and aggregation (Ahmed et al., 2020). Usually, nano-formulations with ZP values around ±30 mV are considered stable. All BLs showed a negative surface charge as ZP ranged from −15.13 ± 2.03 to − 35.11 ± 1.56 mV, indicating that the different formulations had a sufficient surface charge to prevent aggregation and confer BL stability.

As revealed from Table 2, EE% ranged from 81.08 ± 1.7 to 96.98 ± 1.4. Generally, the use of CH results in a higher proportion of EE % because it can efficiently reduce the permeability of the lipid bilayer, and hence drug leakage is inhibited, which translates into enhanced drug encapsulation (Tefas et al., 2017).

### 3.3. *In-vitro* drug release and release kinetics

**Figure 2.**
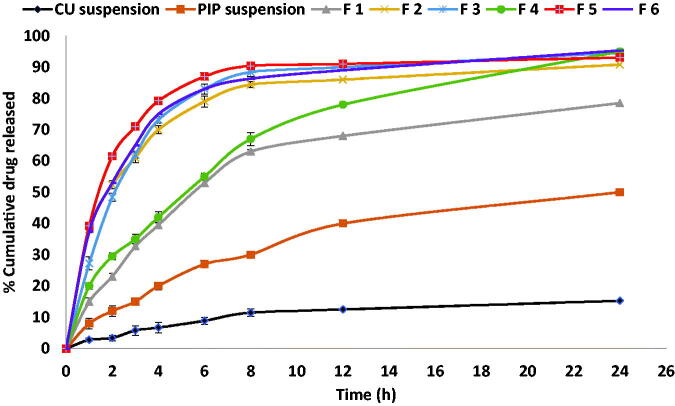
*In-vitro* release profiles of synthesized PIP from different prepared BLs compared to parent CU suspension and synthesized PIP suspension.

*In-vitro* drug release profiles of synthesized PIP from BLs and synthesized PIP suspension compared to release profile of CU suspension are demonstrated in Figure 2. PIP amount released was found to be significantly different among the tested formulae (*p* < .0001 by one-way ANOVA). The Release rate of PIP from BLs was significantly higher (*p* < .0001) than those from PIP or CU suspension. It was worthy to notice that the release rate of parent CU from CU suspension was significantly too lower than the release rate of synthesized PIP. These results might confirm that synthesized PIP had an enhanced solubility rather than parent CU. As expected, pure synthesized PIP release was relatively higher after 1 h compared to CU suspension indicating enhanced solubility.

Abbas et al. and Ribeiro et al. demonstrated that drug nano-encapsulation can enhance the release by increasing water solubility (Ribeiro et al., 2012; Abbas et al., 2021). All tested vesicles released PIP over 24 h. the prepared BLs released about 12–35% in the first 1 h and nearly 45–88% was released after 8 h, and continued up to 24 h. As depicted from Figure 2, the release pattern from PIP-loaded BLs was biphasic over the first 8 h, reflecting an initial rapid release of PIP entrapped within the BLs surface, followed by the slower release of PIP entrapped in the BL score (Abbas et al., 2021). The prolonged PIP release from the investigated BLs vesicles might be due to the advantage of using BLs as colloidal particulate nanostructures. They act as drug reservoirs supplying a prolonged release of the entrapped active molecule (Mohsen et al., 2020; Zafar et al., 2021; 2021). Additionally, the use of cholesterol in preparing the vesicles can decrease the permeability or the liberation of the entrapped drug *via* reducing vesicle membrane fluidity (Mohsen et al., 2020).

As Figure 2 illustrates, the release rate from formulations containing 0.1% SC (F1 and F4) was slower compared with formulations composed of higher SC concentrations (0.3 and 0.6%). This may be attributed to the effect of increasing SC concentration on increasing the release rate of the drug from BLs. This is in agreement with the results previously reported by Zafar et al. (2021) who reported that bile salt concentration has a positive effect on drug release (Zafar et al., 2021).

The drug release mechanism from different formulations, CU and PIP suspensions was investigated by fitting the release data to zero, first order, and Higuchi models (Table 3). According to *R*^2^ values, release from PIP, CU suspension, F1, and F4 followed the Higuchi model that suggests that the drug release occurred mainly by diffusion mechanism (Zewail et al., 2021). On the other hand, the first-order model had the highest *R*^2^ value in F2, F3, F5, and F6 indicating that drug release was time-dependent (Soni & Yadav, 2014).

**Table 3.** *In vitro* release kinetics for the prepared PIP loaded BLs.

Release model	Formulation code							
	CU Susp.	PIP Susp.	F1	F2	F3	F4	F5	F6
Higuchi	***R*^2^ = 0.935**	***R*^2^ = 0.952**	***R*^2^ = 0.964**	*R*^2^ = 0.567	*R*^2^ = 0.663	***R*^2^ = 0.963**	*R*^2^ = 0.663	*R*^2^ = 0.583
First order	*R*^2^ = 0.760	*R*^2^ = 0.840	*R*^2^ = 0.868	***R*^2^ = 0.945**	***R*^2^ = 0.99**	*R*^2^ = 0.90	***R*^2^ = 0.977**	***R*^2^ = 0.975**
Zero order	*R*^2^ = 0.447	*R*^2^ = 0.690	*R*^2^ = 0.283	*R*^2^ = 0.548	*R*^2^ = 0.689	*R*^2^ = 0.38	*R*^2^ = 0.687	*R*^2^ = 0.671

The bolded and underlined values are the highest values of correlation coefficient (R2) representing the kinetic model that best fit the drug release mode of each formula.

### 3.4. Experimental design analysis

Full factorial designs are utilized for studying the effect of different independent variables that might affect the characteristics of the prepared PIP-loaded BLs drug delivery system. As mentioned before, 2^1^.3^1^ full factorial design was used to determine the possible effects of the chosen independent variables and measure their effect on different responses. The yielded non-linear model for EE%, Zp and PS was found to have a significant *p*-value <.0001. As depicted in Table 4, the model F-value of R_1_, R_2_, and R_3_ were found to be 457.84, 11,429.77, and 13,799.5, respectively, which implies the model is significant.

**Table 4.** Summary of 2^1^.3^1^ full factorial design results for prepared PIP loaded BLs.

Response	*SD*	*R* ^2^	Adjusted *R*^2^	Predicted *R*^2^	Adequate precision	Model *F*-value
R_1_: EE(%)	0.45	0.9957	0.9835	0.9917	55.342	457.84
R_2_: ZP (mV)	0.10	0.9999	0.9998	0.9994	282.408	11,429.77
R_3_: PS (nm)	0.22	1.0000	1.0000	0.9999	978.8	13,799.5

SD: standard deviation.

Adequate precision measures the signal noise, a greater ratio than 4 is required and hence this model can be used to navigate space. Additionally, predicted *R*^2^ was calculated to estimate the efficiency of the model to predict a response value. It is worthy to note from Table 3 that, the predicted R^2^ values were 0.9917, 0.9994, and 0.9999 for R_1_, R_2_, and R_3_, respectively, which were in a reasonable agreement with the adjusted *R*^2^ 0.9835, 0.9998, and 1.0000, respectively.

#### 3.4.1. Effect of the independent variables on entrapment efficiency (R_1_, EE%)

**Figure 3.**
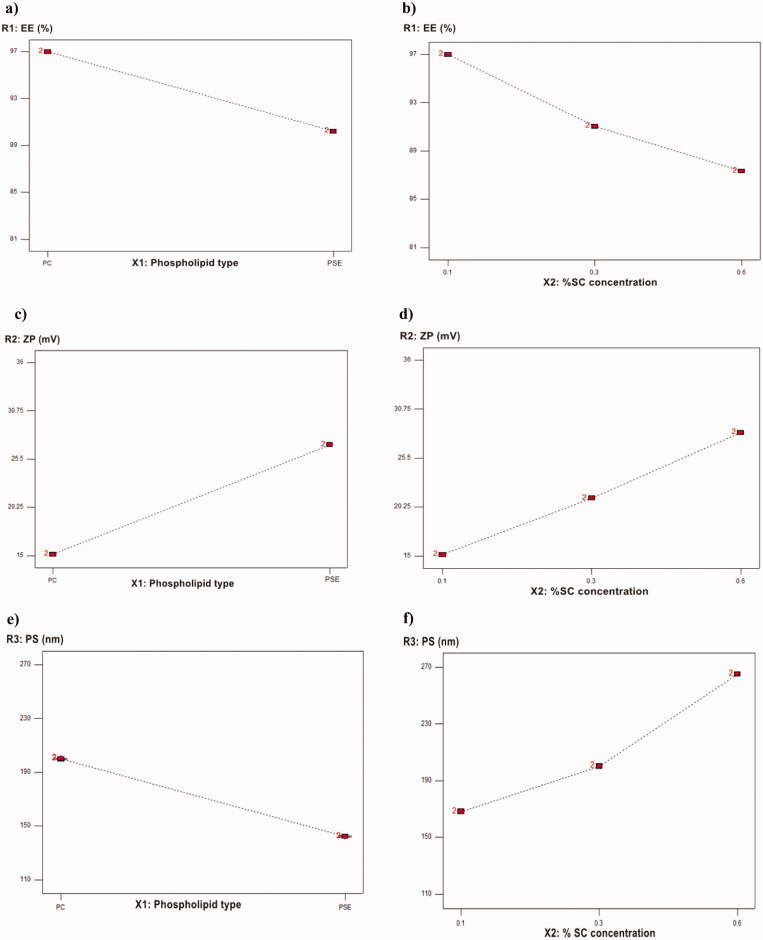
One factor plot of: (a) the effect of X_1_ on R_1_, (b) the effect of X_2_ or R_1_, (c) the effect of X_1_ on R_2_, (d) the effect of X_2_ or R_2, (_e) the effect of X_1_ on R_3_, (f) the effect of X_2_ or R_3_.

**Figure 4.**
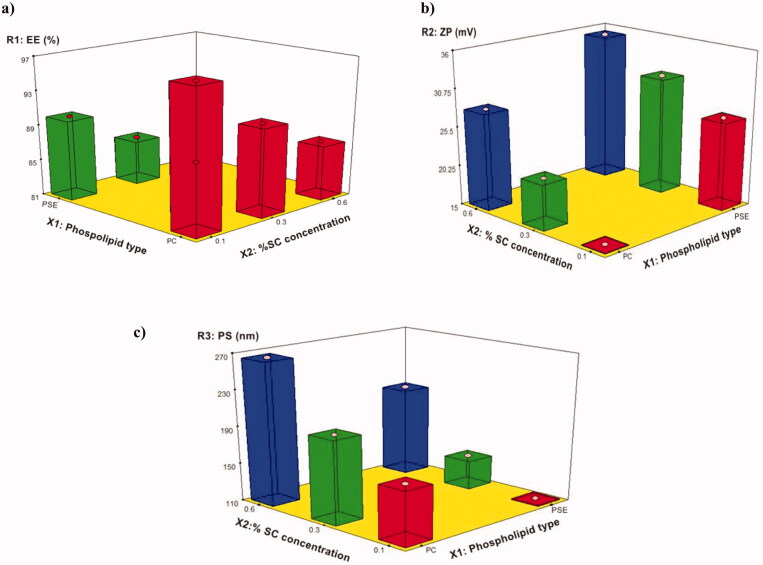
3D surface plot of: (a) the effect of X_1_ and X_2_ on R_1_, (b) the effect of X_1_ and X_2_ or R_2_, (c) the effect of X_1_ and X_2_ on R_3_.

The ability of the prepared BLs to entrap a significant amount of PIP is important for its prospective use as an oral delivery system for PIP. The influence of the phospholipid type (X_1_) and SC concentration (X_2_) on the EE% of the PIP-loaded BLs is graphically illustrated as one factor and 3 D surface plots in Figures 3(a,b), 4(a). With respect to the phospholipid type (X_1_), it was found to positively affect the EE% (*p* < .0001). The EE% of the BLs based on PDC was significantly higher than those prepared with PDS, this results in agreement with the results reported in Miere et al. (2021). In addition, EE% was significantly affected by changing the SC concentration (X_2_), the increase in SC concentration was associated with a simultaneous decrease in EE%. The aforementioned results were in accordance with Al-Mahallawi et al., who stated that bile salts can integrate perpendicularly into the bilayer membrane, disrupt the acyl chains of the lipid matrix, and thereby augment the elasticity and solubility of the drug in the membrane (Al-Mahallawi et al., 2015). Although, higher bile salts concentration synchronously enhances drug solubility in the dispersion medium due to the possible existence of mixed micelles, thereby compromising the EE%. Additionally, at a high concentration of the bile salt, its fluidizing effect on the lipid bilayers of the vesicles could let the entrapped drug escape (Aburahma, 2016; Mohsen et al., 2020).

#### 3.4.2. Effect of the independent variables on zeta potential, ζ (R_2_, ZP)

According to the ANOVA results from Table 3, the model can describe well the effect of different independent variables on ZP response (*p* < .0001). Figures 3(c,d), 4(b) revealed that, ZP was significantly affected by changing phospholipid either PDC or PDS (X_1_). BLs prepared with PDS showed a higher value for ZP compared to those prepared with PDC, demonstrating that these PDS-based BLs are more stable. These results were in accordance with Miere et al. (2021). Moreover, changing SC concentration (X_2_) significantly influenced ZP (*p* < .0001). ZP value for PIP-loaded BLs was significantly increased by increasing the bile salt concentration. These results were counter-intuitive and could be attributed to the bile salt anionic nature, as it would be expected that increasing its concentration could increase the negativity of the prepared vesicles (Abdelbary et al., 2016).

#### 3.4.3. Effect of the independent variables on particle size (R_3_, PS)

The statistical analysis of the obtained data for PS is depicted in Table 3 and Figures 3(e,f), 4(c). Data revealed that phospholipid type (X_1_) was found to have a positive effect on PS. The BLs prepared with PDS showed a lower PS for vesicles compared to those prepared with PDC. This size reduction might be attributed to the lower molecular weight of PDS compared to PDC, which is characterized by being a bulky molecule (Lohr et al., 1995). The same was observed by changing SC concentration(X_2_), it was found to have a significant effect on PS (*p* < .0001). PS had increased by increasing the amount of bile salt. Abdelbary et al. attributed this effect to, the anionic nature of the used bile salt by which high negative ZP values were obtained. Hence, repulsion between the bilayers occurred resulting in a significant increase in the size of prepared BL (Abdelbary et al., 2016).

#### 3.4.4. Formulations optimization

According to Design Expert software, the optimized formula was selected among all the formulae prepared to adopt 2^1^.3^1^ full factorial design. The higher EE% and ZP, and minimum values for PS were set as the criteria to choose the optimized formula. F4 composed of PDS (X_1_) and 0.1% SC (X_2_) showed the highest desirability value of 0.873. F4 showed EE% of 90.21 ± 1.0%, ZP of −27.05 ± 1.08 mV, and vesicle size of 111.68 ± 1.4 nm.

### 3.5. Morphological examination

**Figure 5.**
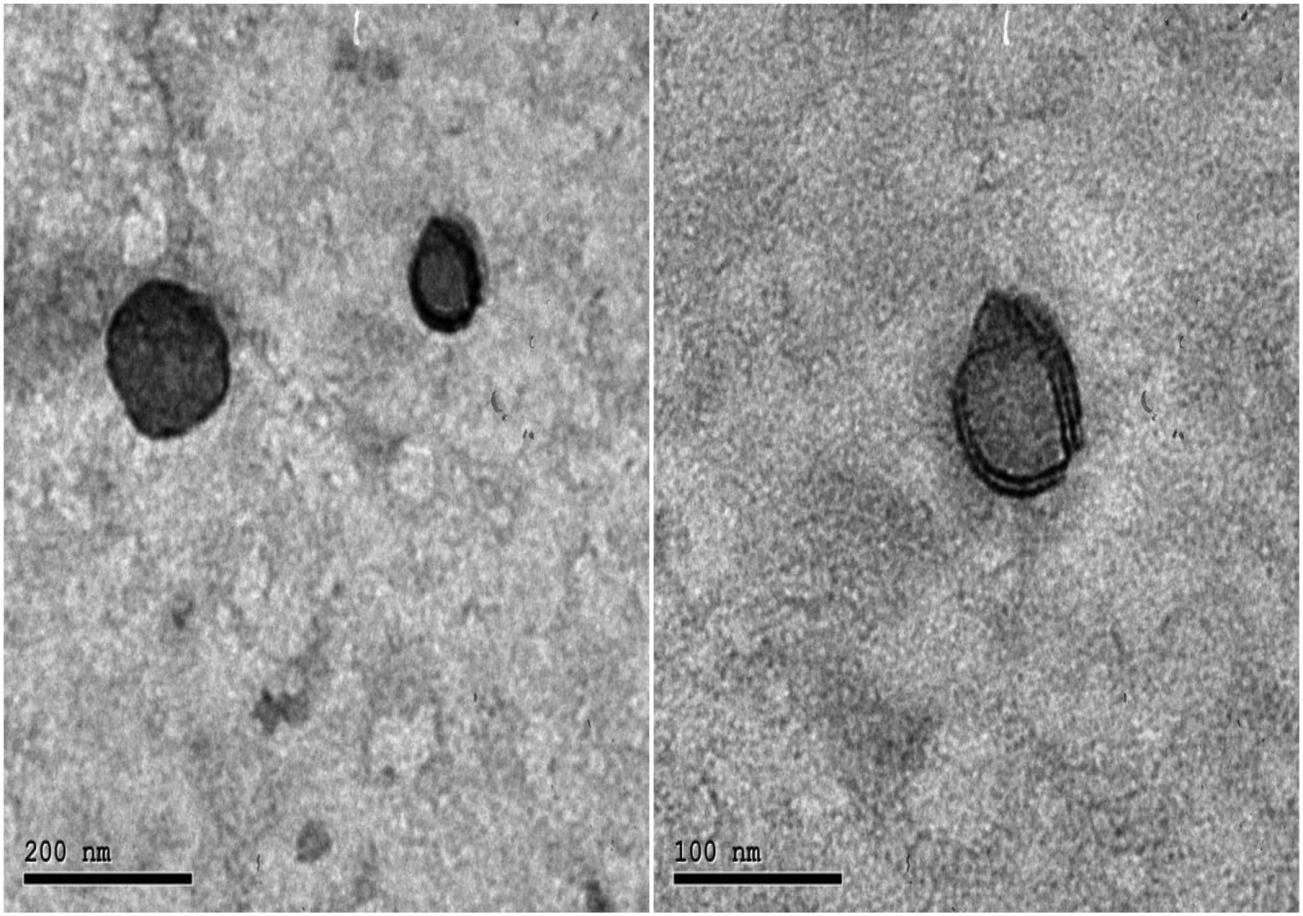
TEM micrographs of optimized PIP loaded BLs (F4) with 80,000 Å magnification.

Morphological examination of the optimized F4 by TEM showed uniform, spherical, non-aggregating vesicles without the formation of drug crystals (Figure 5). The mean observed PS observed by the TEM micrographs was in good agreement with the size analyzed by the Malvern particle size analyzer (Table 2).

### 3.6. Safety assay

**Figure 6.**
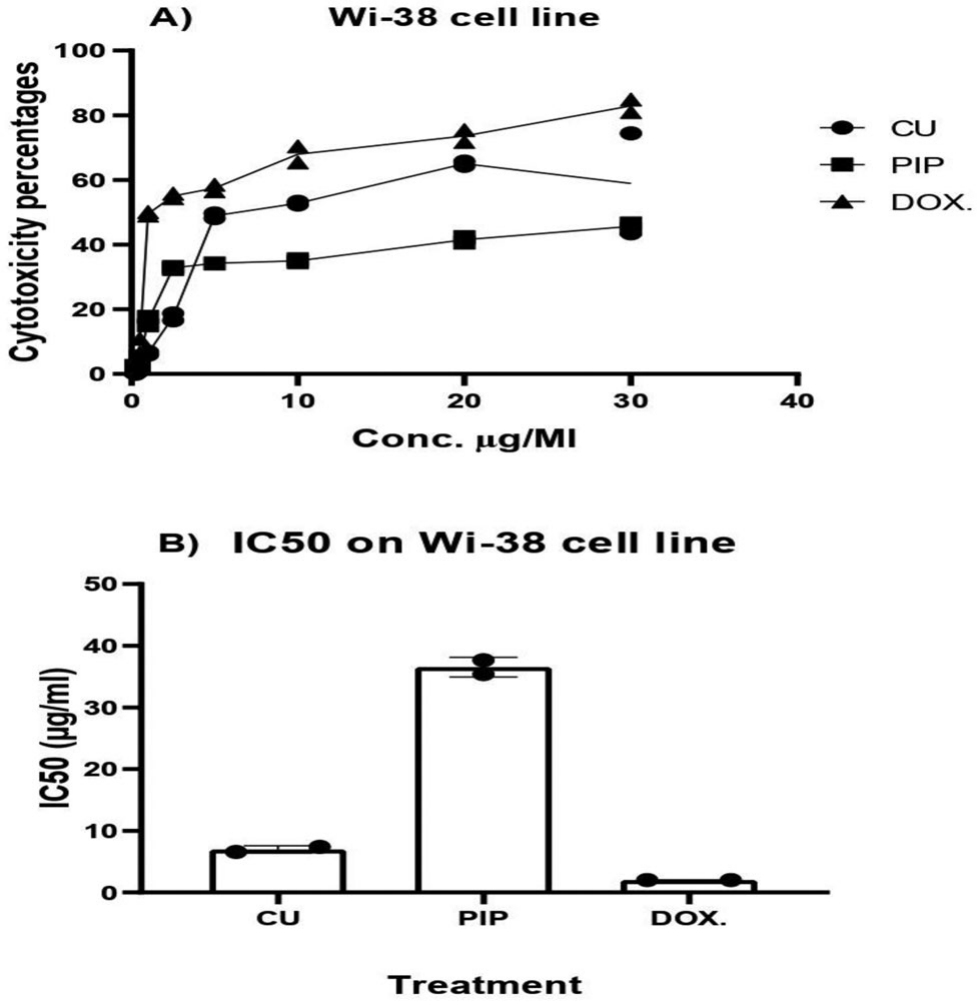
Cytotoxicity assay of CU suspension and PIP loaded BL on Wi-38 cell line.

Different concentrations of CU suspension, PIP-loaded BLs, and doxorubicin (Dox) (from 0.25 to 30 µg/ml) were prepared to compare safety profiles of the tested compounds against a non-cancerous cell line (Wi-38l) (Figure 6(A)). The PIP-loaded BLs recorded the lowest percentage of cell death after cellular treatment with 30 µg/ml (54.6% proliferation%). Furthermore, The PIP-loaded BLs showed the lowest IC50 values with an IC_50_ of 37.64 µg/ml compared to only 6.56 µg/ml for CU suspension and 2.02 µg/ml for Dox (Figure 6(B)). This is the first report that explained the safety usage of PIP-loaded BLs and our results clarified the positive effects of BLs in decreasing the toxic effects of CU on Wi-38 cell line. This could be attributed to the protection action of the BLs content to the cells in addition to over time release of CU from the BLs that could protect the cells from the initial shock treatment of the whole dose at zero time.

### 3.7. *In-vitro* anticancer activity of CU and PIP loaded BLs

**Figure 7.**
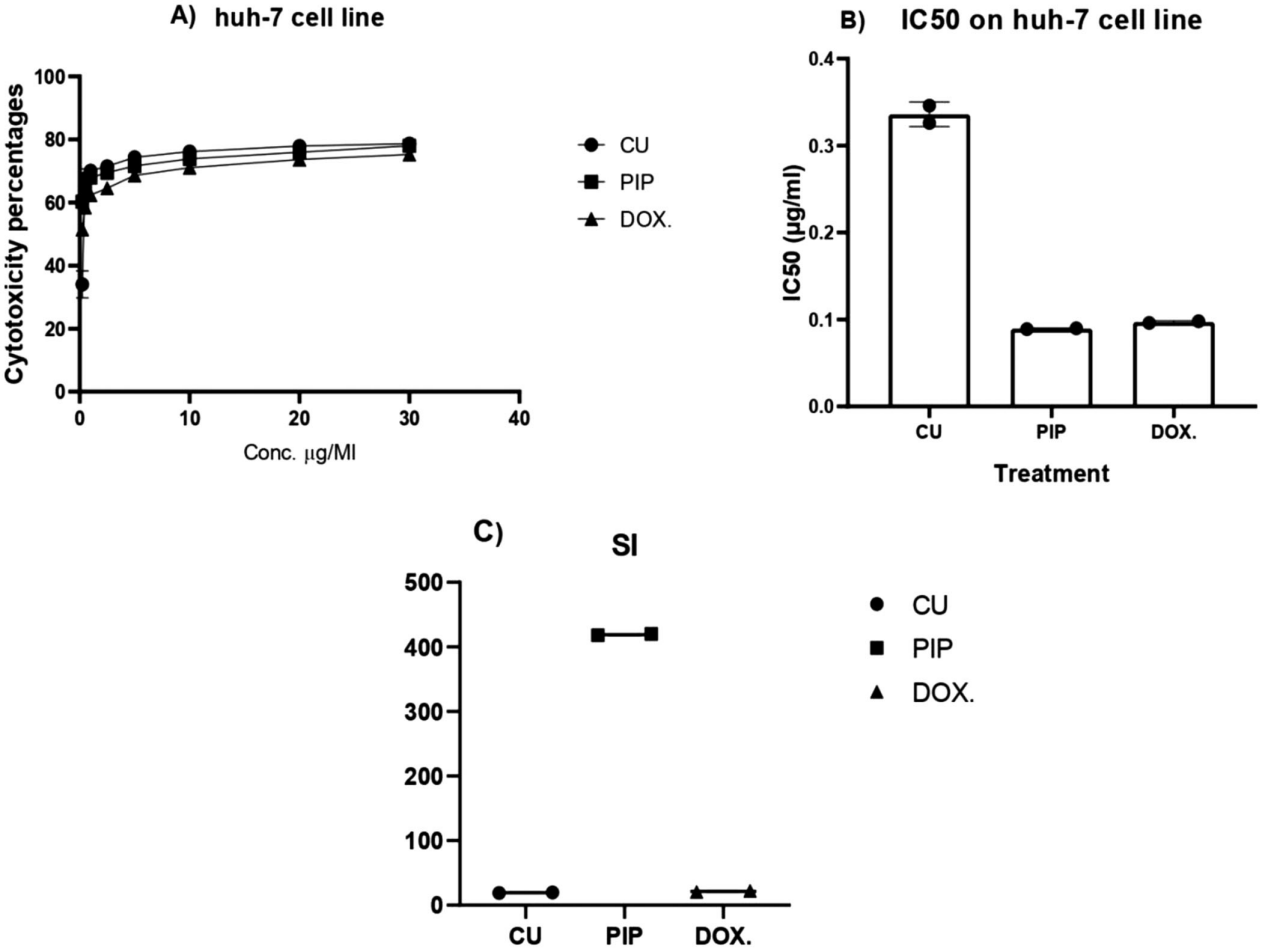
Anticancer and Selectivity index (SI) of CU suspension and PIP loaded BLs against Huh-7 cell line.

The anticancer activities of CU suspension, Dox, and PIP-loaded BLs were compared using Huh-7 cells (Figure 7(A)). The PIP-loaded BLs demonstrated more potent antiproliferative activity than CU suspension (IC_50_ of 0.0895 *vs.* 0.346 µg/ml) and PIP-loaded BLs were roughly as effective as Dox (IC_50_ of 0.097 µg/ml) (Figure 7(B)). The lower antiproliferative activity of CU may be attributable to cryoprotection due to the scavenging of free radicals, such as superoxide anions, nitrogen dioxides, and hydroxyl. However, it was reported that CU can inhibit hepatic cancer cell growth and induce apoptosis (Notarbartolo et al., 2005; Teiten et al., 2010; El-Nassan, 2014; Rafiee et al., 2019) The superior anticancer activity of PIP loaded BLs over CU suspension is likely attributable to chemical modification (Teiten et al., 2010; Salahvarzi et al., 2021) as reported in many other cancer cell lines (Teiten et al., 2010), as well as to the enhanced solubility and stability conferred by encapsulation. Furthermore, PIP-loaded BLs exhibited the highest selectivity index against Huh-7 cells (420.55 *vs.* 18.959 for the CU suspension and 20.82 for Dox) (Figure 7(C)). These results are in accord with those of Mohammed et al., who reported that both CU-loaded nanoparticles and CU suspension reduced chemically induced hepatocellular damage in hepatocellular carcinoma (Mohammed et al., 2021).

### 3.8. Cu and PIP cellular uptake by Huh-7 cells

**Figure 8.**
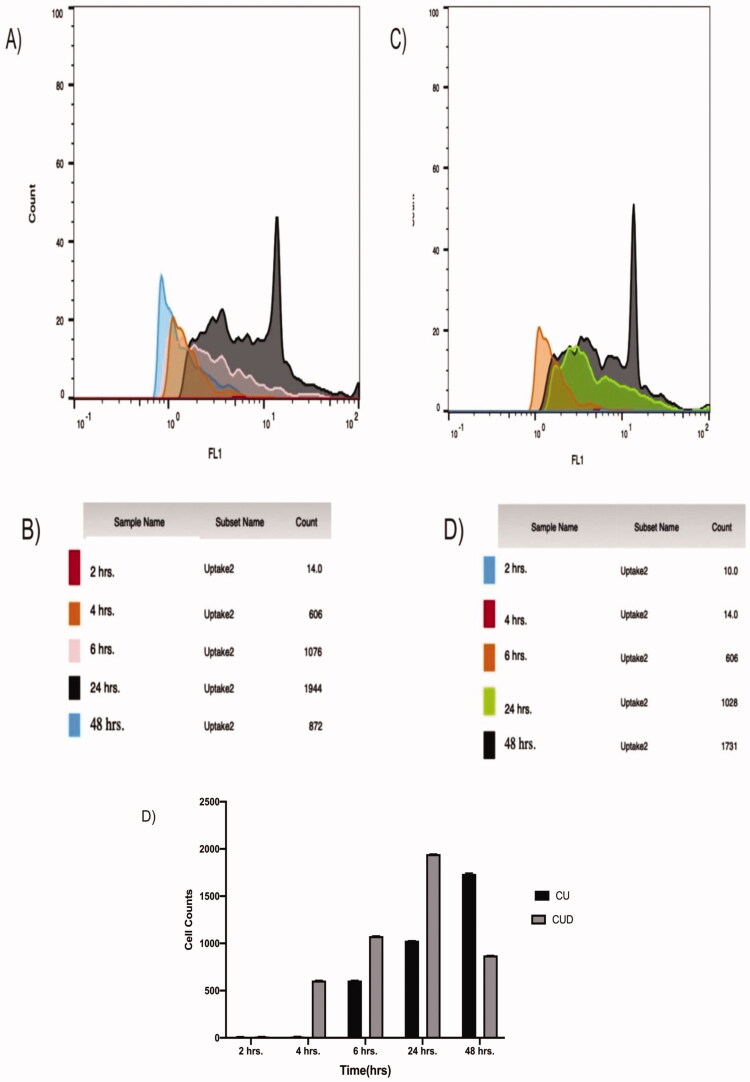
Huh-7 cellular uptake of CU suspension and PIP loaded BLs, (A,B) CUD uptake, (C,D) PIP uptake.

Cellular drug uptake over 24 and 48 h was quantified by flow cytometry following IC_50_ measurements (Figures 8(A–D)). Huh-7 cells accumulated more PIP (1076 positive cells) than CU (606 positive cells) after 6 h. Also, Huh-7 cells accumulated maximum PIP after 24 h (1944 cells) while the maximum CU uptake (1731cells) was not reached even at 48 h.

### 3.9. Quantification of the induced ROS using oxidized DCFDA and flow-cytometry

**Figure 9.**
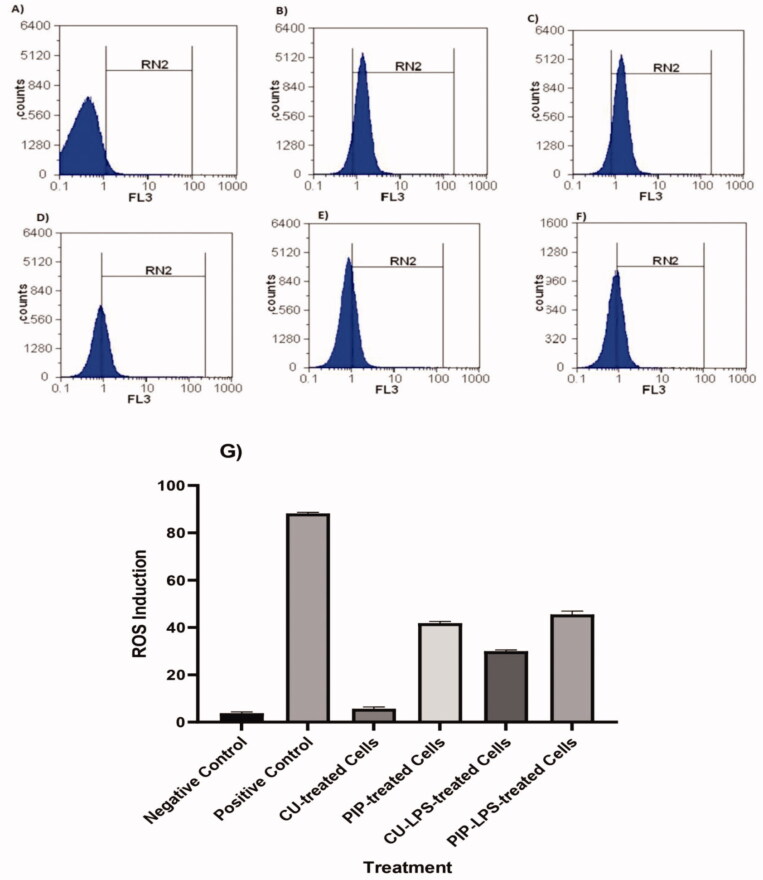
Quantification of the induced ROS in Huh-7 cells, (A) the control untreated cells, (B) LPS-induced cells (positive control), (C) PIP-treated cells, (D) CU-treated cells, (E) PIP-LPS-treated cells, (F) CU-LPS-treated cells, (G) the gatting values of the induced ROS in Huh-7 cells.

The induced cellular ROS in LPS induced PBMCs models and treatment with CU and PIP were quantified using flowcytometry (Figure 9). The obtained results indicated a significant enhancement in the cellular induced ROS in PBMCs after LPS induction (87.87) compared with the un-infected cells (4.16). Also, PIP showed significant ability to induce ROS (41.32) compared with the negative control cells and CU-treated cells (5.11) while in the presence of LPS induction, both PIP and CU showed abilities to reduce the induced ROS from 87.87 to 44.47 and 35.8, respectively.

## 4. Conclusions

The current study supports the use of PIP-loaded BLs as a promising oral drug delivery system for liver cancer treatment. The *in-vitro* release profile showed a biphasic pattern that extended over 24 h with enhanced solubility compared to parent CU. Synthesized PIP-loaded BLs, also demonstrated lower cytotoxicity on non-cancerous Wi-38 cells than parent CU suspension or the clinical anticancer agent Dox. In addition, the formulated BLs displayed superior uptake, selectivity, and anticancer activity compared to CU suspension and Dox. The highly potent antiproliferative effect of synthesized PIP compared to parent CU suggested that this modified compound was more appropriate for nanoencapsulation into BLs as enhanced bioavailability would more likely result in superior antitumor efficacy. Enhanced bioavailability might be furtherly investigated.
